# Renal Medulla is More Sensitive to Cisplatin than Cortex Revealed by Untargeted Mass Spectrometry-Based Metabolomics in Rats

**DOI:** 10.1038/srep44804

**Published:** 2017-03-16

**Authors:** Pei Zhang, Jia-Qing Chen, Wan-Qiu Huang, Wei Li, Yin Huang, Zun-Jian Zhang, Feng-Guo Xu

**Affiliations:** 1 Key Laboratory of Drug Quality Control and Pharmacovigilance (Ministry of Education), China Pharmaceutical University, Nanjing 210009, P. R. China; 2 Jiangsu Key Laboratory of Drug Screening, China Pharmaceutical University, Nanjing 210009, P. R. China; 3 State Key Laboratory of Natural Medicine, China Pharmaceutical University, Nanjing 210009, P. R. China

## Abstract

Nephrotoxicity has long been the most severe and life-threatening side-effect of cisplatin, whose anticancer effect is therefore restricted. Previous pathological studies have shown that both renal cortex and medulla could be injured by cisplatin. Our TUNEL (terminal deoxynucleotidyl transferase-mediated dUTP nick end-labeling) assay results further uncovered that medulla subjected more severe injury than cortex. In order to depict the underlying metabolic mechanism of spatial difference in response to cisplatin, in the present study, mass spectrometry-based untargeted metabolomics approach was applied to profile renal cortex and medulla metabolites of rat after receiving a single dose of cisplatin (2.5, 5 or 10 mg/kg). Eventually, 53 and 55 differential metabolites in cortex and medulla were screened out, respectively. Random forest, orthogonal partial least squares-discriminant analysis and metabolic cumulative fold change analysis revealed that metabolic changes in medulla were more obviously dose-dependent than those in cortex, which confirmed the conclusion that medulla was more sensitive to cisplatin exposure. Furthermore, 29 intermediates were recognized as the most contributive metabolites for the sensitivity difference. Metabolic pathways interrupted by cisplatin mainly included amino acid, energy, lipid, pyrimidine, purine, and creatine metabolism. Our findings provide new insight into the mechanism study of cisplatin-induced nephrotoxicity.

Cisplatin [cis-diamminedichloroplatinum(II)] is an effective antineoplastic agent that was widely applied in the treatment of various types of solid tumors in the past several decades^1^,^2^,^3^. However, due to poor selectivity, cisplatin could cause neurotoxicity, nephrotoxicity, nausea and vomiting, and ototoxicity *et al*. in clinical^4^,^5^,^6^,^7^,^8^,^9^,^10^. As the principal excretory organ for cisplatin, kidney accumulates and retains platinum to a greater degree than other organs^11^,^12^. Therefore, nephrotoxicity has long been the most severe and life-threatening toxicity among these side-effects^13^,^14^. Statistics showed there were about 25–35% patients experienced a significant decline in renal function after receiving a single dose of cisplatin^13^,^15^. The declines manifested clinically as lower glomerular filtration rate, reduced serum magnesium and potassium levels *et al*.^13^,^16^. Research over the past few years has gained significant insights into the mechanisms regarding cisplatin nephrotoxicity, which mainly involved apoptosis, inflammation and oxidative stress *et al*.^17^,^18^,^19^,^20^,^21^,^22^. However, how the toxicity occurs and develops, and how various types of mechanisms are integrated to induce distinct kidney pathology, remain largely unknown.

Metabolomics is an emerging -omics approach that could provide information of holistic and time-dependent metabolic variation in response to xenobiotic interventions^23^,^24^. At present, metabolomics analysis encompasses different strategies depending on the objective of study, namely target analysis of a group of chemically similar metabolites (targeted metabolomics) and global metabolomics profiling (untargeted metabolomics)^25^. Research strategies including sample preparation, instrumental condition, data processing method, and method validation differ from targeted to untargeted metabolomics^26^,^27^. In the past decades, metabolomics has been applied in various research fields such as pharmaceutical and clinical research, food technology and microbiology^28^,^29^,^30^,^31^. In cisplatin nephrotoxicity, metabolomics has also been introduced since 2006^32^,^33^,^34^,^35^,^36^,^37^. The focus of these researches is discovering biomarkers that could be used for the early diagnosis of renal toxicity. Previously, we have investigated the time- and dose-dependent effect of cisplatin nephrotoxicity using untargeted metabolomics approach, and biomarkers with characteristic of time- and dose-dependence were screened out^38^. In these metabolomic studies related to cisplatin nephrotoxicity, serum, plasma and urine were most frequently studied biological matrixes^33^,^34^,^35^,^37^,^38^. However, metabolic alternations in these samples reflect systematic responses originated from all tissues and could not show the specific changes in kidney. Boudonck K. J *et al*. analyzed both serum and kidney samples to discover biomarkers for the early prediction of nephrotoxicity^32^. The experiment was conducted at a very low cisplatin dosage (0.5 mg/kg for 1, 5 or 28 days), which was quite different from the clinical application strategy. What’s more, the effects of dose were not taken into consideration.

When kidney was targeted to conduct metabolomics study of cisplatin nephrotoxicity, the spatial difference was always neglected. Two major regions including the outer cortex and the inner medulla can be visualized if the kidney is bisected from top to bottom^39^. It is generally recognized that cisplatin-induced kidney injury occurs in the cortical part. However, previous morphological study has demonstrated that both cortex and medulla could be impaired by cisplatin as lesions were directly observed in the two parts using light microscopy, transmission electron microscopy and scanning electron microscopy^40^. Moreover, in our pre-experiments, in addition to cortex damage, extremely severe injuries including tubular necrosis, tubular expansion, and tube casts in medulla were observed in pathological sections. What is more interesting is that our TUNEL assay results further uncovered that medulla experienced more severe injury than cortex.

Hence, in order to depict the underlying metabolic mechanism of spatial difference of kidney in response to cisplatin, mass spectrometry-based untargeted metabolomics approach was performed in the present study. The results demonstrated that metabolic profiling of the two parts were distinctly affected due to cisplatin administration. More importantly, medulla showed higher sensitivity to cisplatin than cortex as its metabolic profiling was affected more dose-dependently. Furthermore, 29 metabolites were recognized as the most contributive metabolites for the sensitivity difference by two-step cluster analysis statistical test (CAST). Together our study provides novel insights into the mechanism underlying cisplatin nephrotoxicity from the perspective of metabolomics.

## Results

### Nephrotoxicity induced by cisplatin

Body weight, kidney coefficient, blood urine nitrogen (BUN) and serum creatinine (SCr) of rats in different groups have been reported in our previous study^38^. The results demonstrated that low-dose cisplatin induced slight kidney damage, and medium- or high-dose cisplatin induced significant renal function decline. It should be noted that the initial numbers of rats were 13, 13, 16 and 26 for group C, L, M and H, respectively. Eventually, 2, 3 and 10 rats in group L, M and H died, and the final numbers of animals were 13, 11, 13 and 16 for group C, L, M and H, respectively.

**Figure 1:**
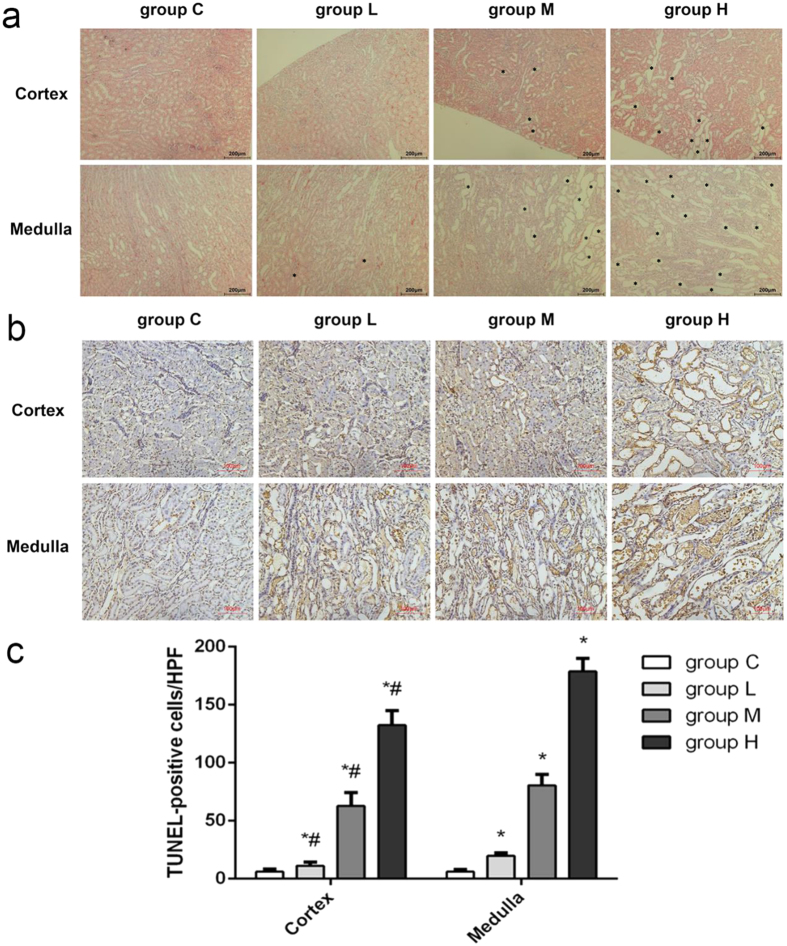
Nephrotoxicity was induced by cisplatin. (**a**) Representative images of H&E staining of cortex and medulla (magnification, 100×), pathological lesions were marked out by asterisks; (**b**) Representative images of TUNEL assay of cortex and medulla (magnification, 200×); (**c**) Histogram of the statistical analysis of positive cells in each high power field. Data are expressed as mean ± SD (n = 5). Mann-Whitney U test, ^#^*p* < 0.05, in comparison with the corresponding group in medulla; *p < 0.05, in comparison with control group.

Not only the macroscopic indicators but also microscopic morphology were significantly changed after cisplatin administration. Figure 1a showed the representative pathological examination results of cortex and medulla in different groups. No abnormal changes were observed in control group (C), and only slight tubular expansion could be observed in medulla in low-dose cisplatin group (L). But remarkable abnormal histological changes can be observed in both cortical and medullar part in medium-dose cisplatin group (M) and high-dose cisplatin group (H). The damages manifested tubular necrosis, tubular expansion, renal epithelial casts and interstitial infiltration of inflammatory cells.

TUNEL assay was utilized to confirm the occurrence of cell apoptosis in cortex and medulla. As can be seen from Fig. 1b, there were large amount of apoptotic cells in both cortex and medulla of group M and H. Cell apoptosis in cortex and medulla in different groups were statistically analyzed by counting positive cells in high power fields (Fig. 1c). The results indicated that cisplatin induced a dose-dependent kidney damage. More importantly, injury of medulla was more severe than cortex.

### Data quality evaluation of untargeted metabolomics

As can be seen from Figure S1a–d, for both medulla and cortex samples analyzed by gas-chromatography-mass spectrometry (GC-MS) or liquid chromatography-mass spectrometry (LC-MS), quality control (QC) samples in PCA score plot were all clustered very well and generally separated from other groups, indicating good reproducibility of the data.

On the other hand, the relative log abundance (RLA) boxplots for QC samples all had a median value close to zero and a similar range of box between samples for both cortex and medulla analyzed by GC-MS or LC-MS approach (Figure S2a–d), suggesting small coefficient of variations within QC samples. Variation in retention times of the 10 representative metabolites in QC samples was shown in Table S1. As can be seen, the variation was less than 0.01 min. Besides, variations in MS response of those metabolites in QC samples were calculated and listed in Tables S2–S5. PCA, RLA plot and variations in retention time and MS response investigation demonstrated that our sample process procedure and detection system were stable throughout the whole experimental period.

### Differential metabolites screened out in cortex and medulla

**Figure 2:**
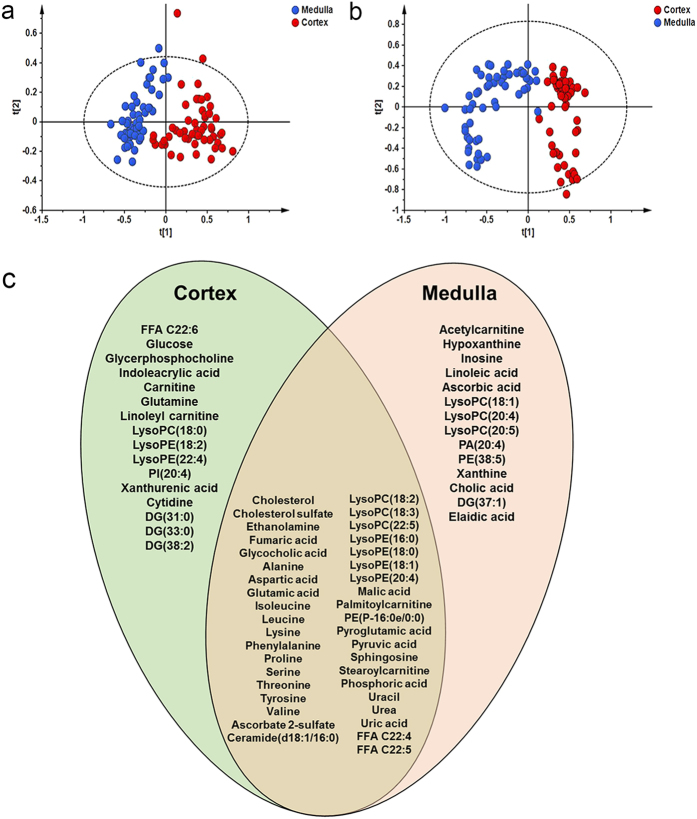
Differential metabolites were screened out in cortex and medulla. (**a**) PCA score plot for all cortex (red circles) and medulla (blue circles) samples based on GC-MS data, model parameters were R2X = 0.799 and Q2 = 0.754; (**b**) PCA score plot of all cortex (red circles) and medulla (blue circles) samples based on LC-MS data, model parameters were R2X = 0.672 and Q2 = 0.552; (**c**) Venn diagram of differential metabolites screened out in cortex and medulla.

In order to test the overall difference between cortical and medullar part, samples from the same part (including four different groups i.e. C, L, M, and H) were combined and used to perform unsupervised PCA first. As Fig. 2a,b showed, cortical and medullar samples were clearly separated in PCA score plots, suggesting that these two parts were truly isolated in the sample collection procedure, and the separation technique was reliable.

Orthogonal partial least squares-discriminant analysis (OPLS-DA) was performed to discriminate differences between C, L, M, and H groups (Figure S3). After differential features screening and metabolite identification, 53 metabolites in cortex and 55 in medulla were screened out, respectively (the detailed information of metabolites were listed in Tables S2–S5). These differential metabolites could be divided into three categories, i.e., 39 common metabolites screened out in both cortex and medulla, 14 metabolites screened out especially in cortex and 16 metabolites characteristically screened out in medulla (Fig. 2c).

### Random forest (RF) revealed sensitivity difference of cortex and medulla

**Figure 3:**
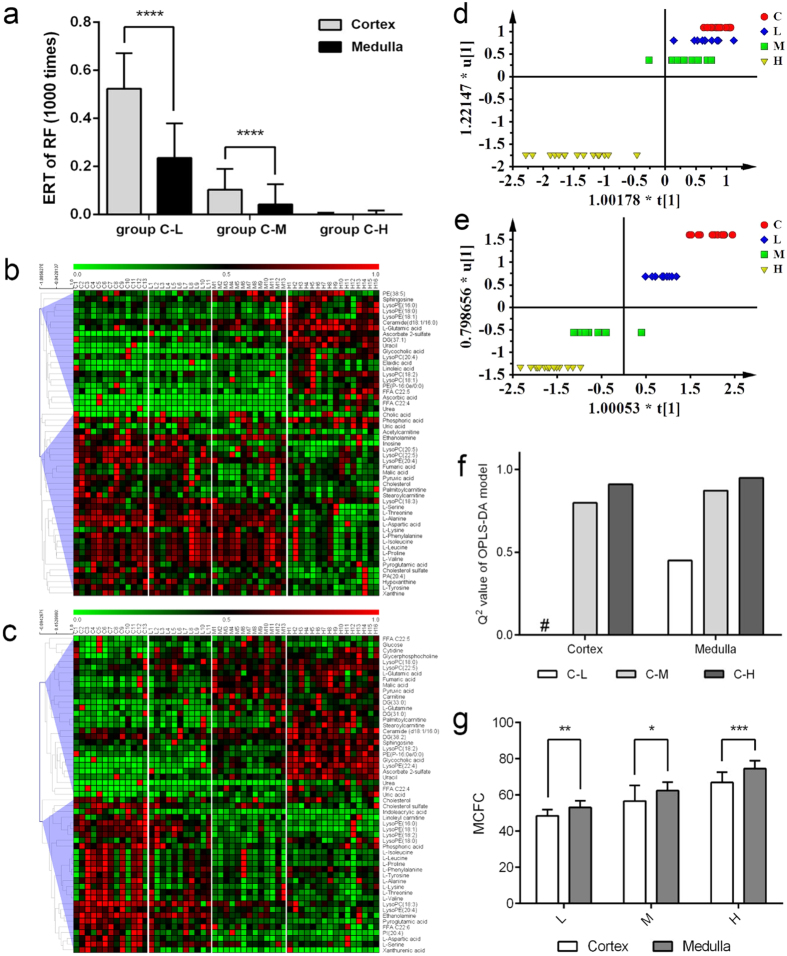
Multiple methods were used to analyze the metabolic sensitivity of renal cortex and medulla to cisplatin dosing. (**a**) Histogram of ERT of RF conducted between control and each cisplatin-treated group; (**b**) Heat-map of differential metabolites screened out in cortex; (**c**) Heat-map of differential metabolites screened out in medulla; (**d**) OPLS-DA score plot constructed based on differential metabolites screened out in cortex; (**e**) OPLS-DA score plot constructed based on differential metabolites screened out in medulla; (**f**) Histogram of Q2 of OPLS-DA models constructed between control and each cisplatin group; (**g**) MCFC of group L, M and H. Data are expressed as mean ± SD. ^#^Negative value. Mann-Whitney U test, ^*^p < 0.05, ^**^p < 0.01, ^***^p < 0.001, ^****^p < 0.0001. Color code in heat-map: red color indicates the highest metabolite concentration and green indicates the lowest.

RF was applied to get a general view of the sensitivity difference between cortex and medulla. One thousand RF models were constructed between group C and each cisplatin-treated group respectively and the error rates of test set (ERTs) were summarized to evaluate the similarity between groups. For example, the error rates of cortex or medulla between group C and H converged to zero, which indicated that metabolic differences between the two groups were obvious. On the contrary, the poor results of cortex in group C and L suggested that the two groups were similar since most of the models were unable to classify samples correctly. Then, as can be seen from Fig. 3a, ERTs of group C vs. L, C vs. M in cortex were significantly higher than those in medulla, while ERTs of group C vs. H in cortex and medulla were similar. These results indicated that metabolic profiling of group L and M were more similar with group C in cortex than medulla, *i.e.*, metabolic profiling of medulla was more susceptible to be affected by cisplatin administration.

### Sensitivity difference was visualized by heat-map

As all detected variables were compared by RF, in order to verify sensitivity difference between cortex and medulla from the perspective of discriminative metabolites, heat-maps were constructed based on identified differential metabolites (53 in cortex, 55 in medulla). From Fig. 3b we can see that concentrations of differential metabolites in cortex were barely influenced by low- or medium-dose cisplatin, while significantly up- or down-regulated by high-dose cisplatin. At variance, change trends of differential metabolites in medulla was dose-dependent (see Fig. 3c).

### Sensitivity difference was validated by OPLS-DA score plot

Inner relation of variable X (differential metabolites) and Y (groups) was discriminated by OPLS-DA. The horizontal axis represented the first principal component and vertical axis the first grouping information. Thus, degree of dispersion of the four groups in the two directions could be used to visualize inter-group differences. As can be seen from Fig. 3d,e, in cortex group C, L and M were very close in both vertical and horizontal direction but completely separated from group H, while in medulla the four groups were dispersed in both the two directions. That is to say, metabolites in medulla were dose-dependently changed and more sensitive to cisplatin dosage.

### Sensitivity difference was quantitatively evaluated by Q^2^ of two-group OPLS-DA

While heat-map and OPLS-DA score plot are excellent visualizing methods to depict sensitivity difference, other methods are needed to evaluate the difference quantitatively. Here, Q^2^ from OPLS-DA constructed between control group and each cisplatin group was used as an indicator (Table S6). Histogram of Q^2^ was shown in Fig. 3f. In cortex, Q^2^ of OPLS-DA model for group C and L was negative, while in medulla, it was 0.45. As for the model of group C vs M and group C vs H, Q^2^ in cortex and medulla were 0.798 vs 0.872, and 0.91 vs 0.949, respectively. Under the circumstances of the same number of predictive and orthogonal components, all Q^2^ values were lower in cortex than those in medulla. Thus, models constructed for medulla were more robust, indicating more distinct differences existed between control samples and cisplatin-treated samples. In another word, metabolite profiling alternations was more significant in medulla than cortex in response to cisplatin administration.

### Metabolic cumulative fold change (MCFC) demonstrated accumulative sensitivity difference

Heat-map, OPLS-DA score plot and parameter Q^2^ were constructed or calculated based on all differential metabolites. To investigate if there were still differences in case of common metabolites in the two parts, MCFC was calculated based on 39 common metabolites (Fig. 2c). As can be seen from Fig. 3g, MCFC of group L, M and H in medulla were all significantly higher than that in cortex. Thus, the degree of accumulative metabolic change of medulla was higher than cortex, indicating that metabolites in medulla were more sensitive to cisplatin exposure than cortex.

### Contributing metabolites were screened out by two-step cluster analysis statistical test (CAST)

After validation of sensitivity difference of cortex and medulla in response to cisplatin injection, there must be a query that what metabolites contributed most to the difference. As metabolic changes in medulla were more obviously dose-dependent than cortex, metabolites with good correlation to cisplatin dosage in medulla were thought contributive. To screen out these metabolites, a two-step CAST method was utilized. First, fold changes of metabolites between each cisplatin group and control group were calculated. Then, a union of 108 differential metabolites (53 from cortex, 55 from medulla) was imported to MeV software. After the first step CAST, two main clusters of metabolites were generated and kept. Cluster one included 41 metabolites which showed an increase trend as cisplatin dosages elevated. Cluster two was consisted of 43 metabolites whose concentrations decreased as the dosages increased. The left 24 metabolites had poor correlation with cisplatin dosages were excluded.

**Figure 4:**
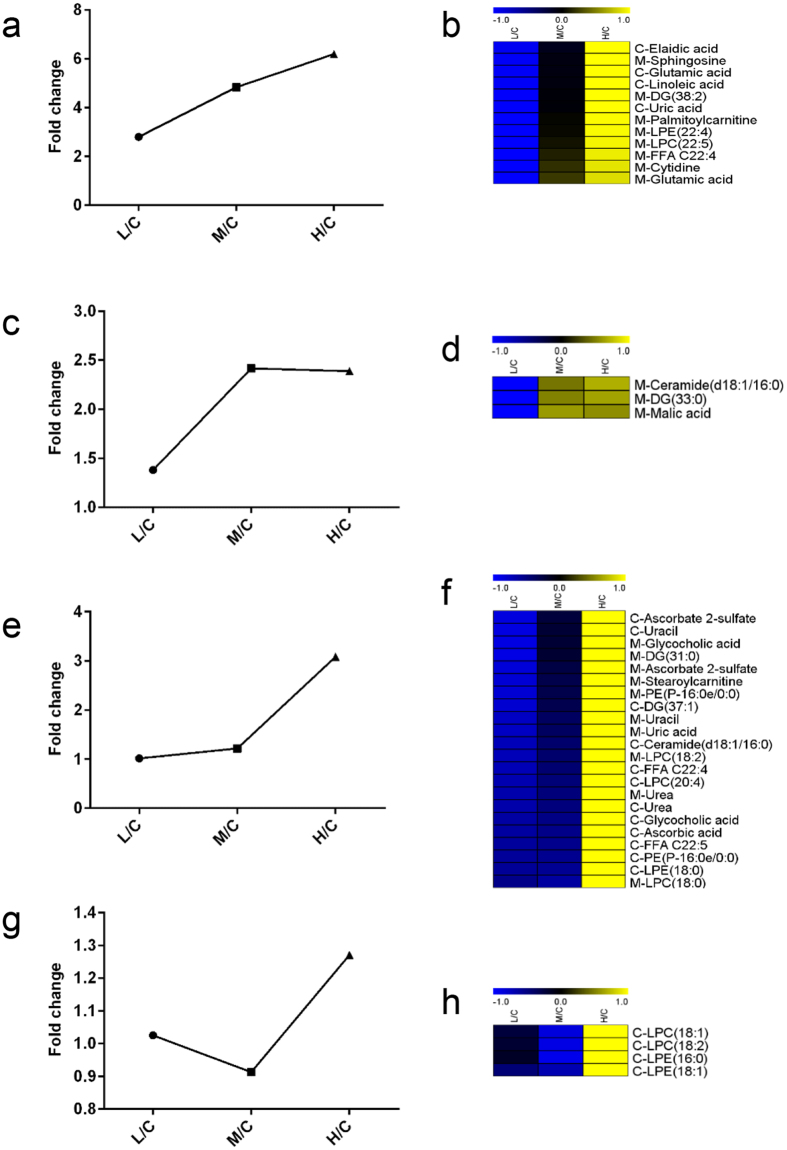
Four sub-clusters were generated within cluster one after the second CAST. (**a**) Representative change trend of metabolites in sub-cluster 1. (**b**) Heat-map of metabolites in sub-cluster 1. (**c**) Representative change trend of metabolites in sub-cluster 2. (**d**) Heat-map of metabolites in sub-cluster 2. (**e**) Representative change trend of metabolites in sub-cluster 3. (**b**) Heat-map of metabolites in sub-cluster 3. (**g**) Representative change trend of metabolites in sub-cluster 4. (**b**) Heat-map of metabolites in sub-cluster 4. Capitalized C before metabolite name means this metabolite was screened out from cortex, and M from medulla.

Then, the second CAST was executed within cluster one and two, respectively. As a result, four sub-clusters were generated from cluster one. Sub-cluster 1 included 12 metabolites showed great correlation with cisplatin dosages, i.e. fold changes gradually increased as the dose elevated. Representative tendency chart was shown in Fig. 4a, and the corresponding heat-map was shown in Fig. 4b. These metabolites may contribute largely to the sensitivity difference as they were very sensitive to cisplatin dosage. In this sub-cluster, 4 metabolites were from cortex, and the left 8 were from medulla. Sub-cluster 2 included 3 metabolites whose concentrations in group M and H were significantly higher than that in group L (Fig. 4c). They were all from medulla (Fig. 4d). Sub-cluster 3 included 22 metabolites, whose fold changes also increased as dosages elevated. However, fold changes of metabolites in group M were similar with group L, while in group H, they were significantly higher than those in group L and M (Fig. 4e). It was indicated that these metabolites were sensitive to high-dose while not to low- or medium-dose cisplatin. In this sub-cluster, 12 metabolites were from cortex and 10 from medulla (Fig. 4f). As shown in Fig. 4g,h, metabolites in sub-cluster 4 had poor correlation with cisplatin dosages were all screened out from cortex.

**Figure 5:**
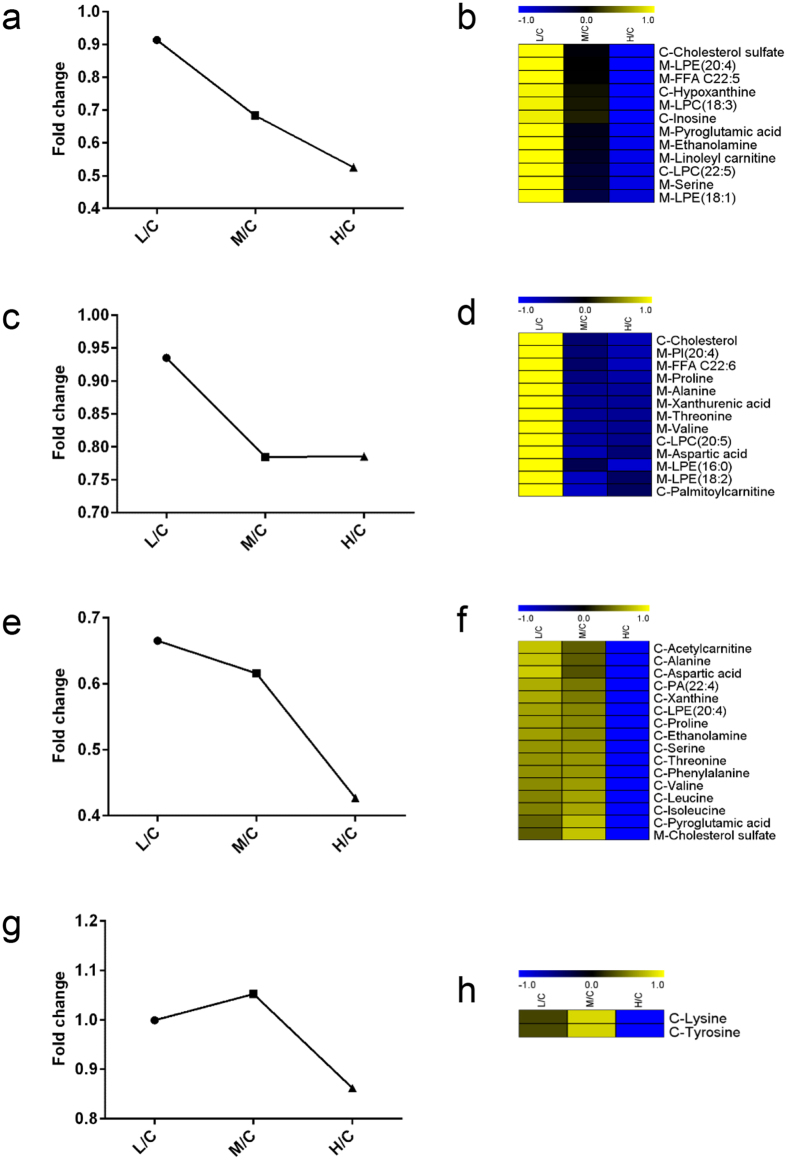
Four sub-clusters were generated within cluster two after the second CAST. (**a**) Representative change trend of metabolites in sub-cluster 1. (**b**) Heat-map of metabolites in sub-cluster 1. (**c**) Representative change trend of metabolites in sub-cluster 2. (**d**) Heat-map of metabolites in sub-cluster 2. (**e**) Representative change trend of metabolites in sub-cluster 3. (**b**) Heat-map of metabolites in sub-cluster 3. (**g**) Representative change trend of metabolites in sub-cluster 4. (**b**) Heat-map of metabolites in sub-cluster 4. Capitalized C before metabolite name means this metabolite was screened out from cortex, and M from medulla.

Similarly, cluster two was further divided into four sub-clusters after the second CAST. As shown in Fig. 5a, metabolites in sub-cluster 1 were greatly correlated to cisplatin dosages, 4 of which were from cortex and 8 from medulla (Fig. 5b). These metabolites were also speculated to be contributive to the sensitivity difference. In sub-cluster 2, there were 13 metabolites, whose fold changes in group M and H were significantly lower than those in group L (Fig. 5c), indicating that they were more sensitive to medium- and high-dose cisplatin. Of metabolites in sub-cluster 3, only 3 were from cortex, and the left 10 were from medulla (Fig. 5d). Sub-cluster 3 included 16 metabolites. In group H, fold changes of these metabolites were significantly lower than those in group L and M, indicating that those metabolites were only affected by high-dose cisplatin and lacking sensitivity to low- and medium-dose cisplatin (Fig. 5e). 15 of 16 metabolites in this sub-cluster were from cortex (Fig. 5f). The left 2 metabolites without good correlation with cisplatin dosages were included in sub-cluster 4 (Fig. 5g), and they were all from cortex (Fig. 5h).

Altogether, most of the metabolites sensitive to both medium- and high-dose cisplatin were from medulla (Figure S4a), while the majority metabolites that only sensitive to high-dose cisplatin and all metabolites poorly correlated with cisplatin dosages were derived from cortex (Figure S4b). Totally 29 metabolites greatly correlated with cisplatin dosages or sensitive to both medium and high-dose cisplatin in medulla were thought contributed most to the sensitivity difference. These metabolites were sphingosine, diacylglycerols (DG) including DG(38:2) and DG(33:0), lyso-phosphatidylcholines (LysoPC) including LysoPC(22:5) and LysoPC(18:3), lyso-phosphatidylethanolamines (LysoPE) including LysoPE(22:4), LysoPE(18:1), LysoPE(20:4), LysoPE(16:0) and LysoPE(18:2), phosphoinositol (PI) like PI(20:4), free fatty acids (FFA) including FFA C22:4, FFA C22:5 and FFA C22:6, ceramide(d18:1/16:0), ethanolamine, palmitoylcarnitine, linoleylcarnitine, cytidine, malic acid, glutamic acid, pyroglutamic acid, proline, alanine, threonine, valine, aspartic acid, serine and xanthurenic acid.

### Altered pathways related to cisplatin nephrotoxicity

**Figure 6:**
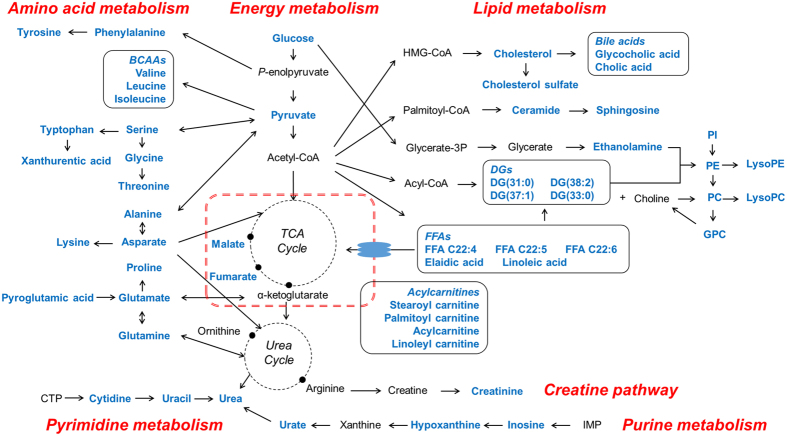
Metabolic pathways interrupted by cisplatin. Metabolite names appear in blue fonts are differential metabolites identified in the present study. Abbreviations in this figure: TCA, tricarboxylic acid; DGs, diacylglycerols; LysoPC, lyso-phosphatidylcholine; PC, phosphatidylcholine; LysoPE, lyso-phosphatidylethanolamines; PE, phosphatidylethanolamines; PI, phosphoinositol; FFAs, free fatty acids; BCAAs, branched-chain amino acids; GPC, glycerophosphocholine; IMP, inosine monophosphate; CTP, cytidine triphosphate.

As can be seen from Fig. 6, several metabolic pathways were affected by cisplatin including amino acid, energy, lipid, pyrimidine and purine metabolism, as well as creatine pathway. Of these metabolic pathways, lipid and amino acid metabolism were the most perturbed ones.

## Discussion

Cisplatin is taken into the cells primarily by organic cation transporter 2 (OCT2) of the proximal tubules and then transported to the apical site where it is bio-activated into a more potent metabolite (cysteinyl-glycine-conjugates) by gamma-glutamyl transpeptidases that are present in the proximal tubules. Then cysteinyl-glycine-conjugates are further metabolized to cysteine-conjugates by aminodipeptidases, also expressed on the surface of the proximal tubule cells. The cysteine-conjugates are then transported into the proximal tubule cells, where they are further metabolized by cysteine-S-conjugate beta-lyase to highly reactive thiols^41^,^42^. Previous mechanism studies of cisplatin nephrotoxicity showed that the most damaged region is renal proximal tubule^12^,^43^, which is generally thought to be located in cortex. But actually, renal proximal tubule could be further divided into three segments, i.e. S1, S2 and S3 segment. The S1 and S2 segments make up the pars convolute and are situated in cortex, while the S3 segment locates in the outer stripe of medulla and in medullary rays. Study found that OCT2 which responsible for transporting cisplatin was mainly located in the S2 and S3 segment^44^. In addition, Dobyan’s study showed that the S3 segment accumulated the highest concentration of cisplatin and is the most prominent damaged segment after cisplatin administration^40^. The findings in the present study also demonstrated that medulla would be more severely damaged than cortex under the same dosage of cisplatin, which might be a suggestion to researchers that more attentions should be paid on the medullar part when mechanism studies related to cisplatin-induced nephrotoxicity conducted. Besides, for the time being more attentions was paid on cortex in cisplatin nephrotoxicity, and medulla seemed to be ignored. In *in vitro* studies, renal cortical slices, primary cells or passage cells (including NRK-52E, normal rat kidney proximal cell; LLC-PK1, porcine kidney epithelial cell line; HK-2, human renal proximal tubule epithelial cell) derived from cortex were most routinely used for cytotoxicity test, while no cell model originated from medulla was established and adopted^45^,^46^,^47^,^48^,^49^. In the future, cell lines derived from medullar part could be established for cytotoxicity researches.

Previous studies revealed that during cisplatin-induced acute renal failure, there was a significant reduction in proximal tubule of fatty acid oxidation, which lead to the accumulation of fatty acids^35^,^36^. In this study, fatty acids in cortex and medulla were observed up-regulated. In addition, DGs were also found elevated. DGs are glycerides consisting of two fatty acid chains covalently bonded to a glycerol molecule through ester linkages. They also serve as precursors to triacylglycerol (TG) by the addition of a third fatty acid to the DG under the catalysis of diglyceride acyltransferase. A ^1^H-NMR based metabolomics study discovered increased TGs after cisplatin exposure^18^. Taken together, cisplatin administration exerted great influence on lipid metabolism in kidney. Inhibition of lipid accumulation in kidney may have the potential to alleviate cisplatin nephrotoxicity. It has been reported that precursors like PPAR-α possessed the ability of decreasing fatty acid and TG accumulation, and cisplatin nephrotoxicity was then reduced^35^,^36^. This finding suggested that inhibiting lipid accumulation might be a target to ameliorate cisplatin-induced nephrotoxicity. Ceramide and its metabolite sphingosine were found up-regulated in both cortex and medulla in the present study. Ceramide has been demonstrated plays important role in cell apoptosis by acting on several putative and direct targets like ceramide activated protein kinase, cathepsin D, and serine/threonine protein phosphatase 1 and 2A^50^,^51^. The relationship of ceramide and cell apoptosis has been identified in many other disease models. Though there is no direct evidence, we speculate that ceramide might have significant effect on cisplatin-induced renal injury.

In case of amino acid metabolism, it has been reported that one of the strongest responses induced by nephrotoxin was a dramatic decrease in amino acids in kidney^32^. Previous metabolomics studies have demonstrated the significant changes of amino acids in cisplatin-induced nephrotoxicity. In our study, except for glutamic acid was found increased in the two parts, other amino acids were down-regulated in both cortex and medulla. In mammals, about 99% of filtered amino acids are reabsorbed in the proximal tubule. However, under nephrotoxic conditions, amino acid excretion often increase because of impaired reabsorption by the renal tubules, increased cellular turnover, or increased permeability of the glomerular membranes. As a result, contents of amino acids in kidney obviously decreased. Preclinical studies demonstrated that the supplement of amino acids could ameliorate acute kidney injury^52^,^53^, which indirectly demonstrated the deficiency of amino acids in kidney during the development of nephrotoxicity. Besides, other urinary metabolomic studies in cisplatin-induced renal toxicity revealed that amino acids in urine were drastically increased^32^,^35^,^54^, which also in accordance with the findings in the present study. More and more attentions had been drawn on the protective effects of endogenous metabolites. Glutamine has been demonstrated with the ability of attenuating cisplatin nephrotoxicity by decreasing cisplatin accumulation in rats and HK-2 cells^55^. Glycine and L-arginine were also proved with protective effect against cisplatin-induced nephrotoxicity in rat renal cortical slices^56^. In future studies, we plan to investigate whether other metabolites have protective effects on cisplatin renal injury.

## Methods

### Chemicals and reagents

Cisplatin injection was purchased from Haosen Pharmaceutical (Lianyungang, China). Chemicals used in GC-MS derivatization process including O-Methoxyamine hydrochloride, N-methyl-N-trifluoroacetamide (MSTFA) and pyridine were purchased from Sigma-Aldrich (St Louis, MO, USA). Standard compounds used for metabolite identification were also obtained from Sigma-Aldrich. LC-MS grade reagents like methanol, acetonitrile and ethyl acetate were obtained from Merck (Germany). Deionized water was produced using a Milli-Q system (Millipore, Bedford, MA, USA).

### Animal experiment and sample collection

All animal experimental protocols were according to the guide for the care and use of laboratory animals (8^th^ edition) released by the National Research Council of the National Academies, and all experimental protocol was approved by the Animal Ethics Committee of China Pharmaceutical University (License Number: SYXK 2012-0035). Male Sprague-Dawley rats, six to seven weeks old (Nanjing, China), were allowed to acclimatize for a week. All rats were fed with a standard commercial diet while housed in a light- and temperature-controlled room (12/12 h light/dark, 22–25 °C, 45–55% humidity).

At the first day after acclimatization, rats were intravenously administered with a single dose of cisplatin, and the dosages were 2.5 mg/kg (low dose, group L, n = 13), 5.0 mg/kg (medium dose, group M, n = 16) and 10.0 mg/kg (high dose, group H, n = 26). The dosages were made according to converted human dosage (7.75 mg/kg), lethal dose of 50% (LD_50_) in rats (approximately 13 mg/kg), our pre-experiment results and existing literature. Rats in control group (group C, n = 13) was intravenously administered with an equivalent volume of normal saline.

At the seventh day after dosing, rats were sacrificed after blood collection. The sampling time was made combining results of our pre-experiment and existing literature^32^,^33^,^34^,^35^,^37^. The left kidneys were removed, weighted and dissected on an ice plate to separate the cortical and medullar part. All the samples were kept at −80 °C until metabolomics analysis. The right kidneys were removed, weighted and cut into two portions. One portion was fixed in 10% neutral-buffered formalin for hematoxylin and eosin (H&E) staining and another in 4% neutral-buffered paraformaldehyde for TUNEL assay.

### H&E staining and TUNEL assay

After fixed overnight in 10% neutral-buffered formalin, kidneys were dehydrated in alcohol and then embedded in paraffin. Paraffin sections were prepared and stained using standard H&E staining methods. TUNEL assay kit (*In Situ* Cell Death Detection Kit, POD) was purchased from Roche (USA). Briefly, following dewaxing and hydration, kidney sections were digested with proteinase K and labeled with a TUNEL reaction mixture for 60 min at 37 °C in the dark. After the addition of converter and substrate solution, samples were analyzed by light microscopy. H&E staining and TUNEL assay were conducted by a professional pathologist of Nanjing Medical University.

### Sample pretreatment for instrumental analysis

Frozen renal cortex or medulla (20 mg) were firstly placed into pre-cooled 2 mL homogenization tubes containing ceramic beads. Then, pre-cooled methanol was added (10 μL/mg tissue). The samples were homogenized for three times (5.5 m/s for 30 s), with 60 s intervals between homogenization steps. After two centrifugations (14000 rpm, 5 min, 4 °C), the supernatant was removed and named as kidney homogenate. For LC-MS analysis, 125 μL acetonitrile was added to a 25 μL aliquot of the kidney homogenate. The solution was mixed thoroughly and centrifuged twice (14000 rpm, 5 min, 4 °C), and the supernatant was removed for LC-MS analysis. For GC-MS analysis, 100 μL methanol was added to a 10 μL kidney homogenate. The next derivation steps referred to our previous studies^16^,^29^.

### GC-MS analysis of kidney samples

GC-MS analysis was carried out on Shimadzu GCMSQP2010 Ultra (Ultra GC-Q/MS; Shimadzu Inc., Kyoto, Japan) equipped with a fused silica capillary column (Rtx-5MS; 30 m × 0.25 mm, 0.25 μm, Restek, USA). Helium was employed as the carrier gas the flow rate was set at 1.0 mL/min. The programmed oven temperature was started at 70 °C for 2 min, followed by an increase to 320 °C at the rate of 10 °C/min and maintained at 320 °C for 2 min. The temperature of the injector and ion source were maintained at 250 °C and 200 °C, respectively. Electron impact mode with the energy of 70 eV was employed for the ionization. Data acquisition was performed in full scan mode with the mass to charge ratio (m/z) from 45 to 600. The injection volume was 1 μL, and the split ratio was 50:1. GCMS Solution software (Shimadzu Inc., Kyoto, Japan) was used for auto-acquisition of total ion chromatograms (TICs) and fragmentation patterns.

### LC-MS analysis of kidney samples

LC-MS analysis was performed on a Shimadzu Prominence series ultra-fast liquid chromatography (UFLC) system coupled to ion trap/time-of-flight hybrid mass spectrometry (IT/TOF-MS, Shimadzu, Japan). Chromatographic separation was achieved by a Phenomenex Kinetex C18 column (100 × 2.1 mm, 2.6 μm; Phenomenex, USA). The column temperature was held at 40 °C. The gradient elution involved a mobile phase consisting of (A) 0.1% formic acid in water and (B) acetonitrile, with a programmed gradient as follows: linear gradient from 5% B to 95% B, 0–20 min; maintained with 95% B in 10 min. The flow rate was at 0.4 mL/min and the injection volume was 5 μL. Electrospray ionization method was applied. The mass range of the full scan was set from m/z 100 to 1000 in both positive and negative ion mode, with interface voltage of 4.5 kV and −3.5 kV, respectively. The curved desorption line and heat block temperature were both 200 °C. The detector voltage of the TOF analyzer was 1.65 kV. Nitrogen was used as the nebulizer and drying gas, set at a constant flow rate of 1.5 L/min and 10 L/min, respectively. In the tandem mass spectrometry experiments, argon was employed as the collision gas, and the collision energy was set at 10, 20, 30, 40, 50 or 60 eV. LCMS Solution software (Shimadzu Inc., Kyoto, Japan) was used for auto-acquisition of TICs and fragmentation patterns.

### Data quality evaluation

During the instrument analysis, all samples from control and cisplatin-treated rats were randomized in order to avoid inter-batch differences. QC samples were prepared by pooling equal aliquot of each kidney homogenate and treated congruously with real samples. The first 10 QCs were tested in both GC-MS and LC-MS before the real sample analysis to stabilize the analytical system and removed before data processing. In order to monitor the robustness of sample preparation and the stability of instrument analysis, QC samples were intermittently injected through the analytical batch. Ten real samples were inserted with one QC sample in LC-MS analysis, and six real samples were inserted with one QC sample in GC-MS analysis.

Data quality evaluation strategy included the following aspects: 1) unsupervised pattern recognition method PCA was constructed based on all QC samples and cortex or medulla samples (SIMCA-P software, Version 13.0, Umetrics, Sweden). PCA score plot was used to evaluate the data quality; 2) within-group RLA plot based on all extracted features was utilized to evaluate the coefficient of variations of QC samples (R program)^57^,^58^. Within-group RLA plot was calculated by subtracting median value within- or across-group for each metabolite after log transformation. For within-group RLA, the boxplot of features of each QC sample would have a median value close to zero and a similar range of box between samples if the coefficient of variations in QC samples was small; and 3) retention time variation for representative metabolites were investigated in QC samples in both GC-MS and LC-MS analysis.

### Data preprocessing, analysis and differential features screening

Data extraction was performed by Profiling Solution Software (Shimadzu Inc., Kyoto, Japan). After the data pretreatment^59^,^60^, a matrix containing grouping information, sample names, retention times and normalized peak intensities were obtained. Mass spectrometry total useful signal (MSTUS) method was used for the normalization of signal intensities. OPLS-DA was performed by SIMCA-P software. Features (a feature here was defined as a unique pair of RT and m/z record) were treated as differential if the following conditions were met. First, variable importance in the projection (VIP) value should be greater than 1.0 in OPLS-DA constructed between control and each experimental group. Second, confidence intervals on VIP column plot should be positive. Third, adjusted *p* value of Wilcoxon Mann-Whitney Test and stricter false discovery rate (FDR) correction based on Benjamini-Hochberg method (MeV, Version 4.6.1, http://www.tm4.org/) should be lower than 0.05. After the feature screening process, those differential features were prepared for metabolite identification.

### Metabolite identification

For LC-MS analysis, a comprehensive strategy was used for metabolite identification. Firstly, Pearson correlation analysis was performed to cluster features (i.e., molecule ions, in-source molecular fragments, (de)protonated molecule ions, adducts ions and ^13^C isotopes) originating from the same metabolite. Second, Formula Predictor in LCMS Solution software was utilized to predict the compound formula by the comparison of theoretical and observed m/z values and isotopic patterns. Then, retention time, accurate m/z, and the MS/MS fragmentation of features of interest were compared with those standard compounds available in our lab. Features that did not match with any standard compounds were then compared with those provided by existing literatures and online databases, such as HMDB (http://www.hmdb.ca), METLIN (http://metlin.scripps.edu), LIPID MAPS (http://www.lipidmaps.org) and Mass Bank (http://www.massbank.jp) *et al*. Finally, the confidence of identified metabolites was ranked by referring to the metabolomics standards initiative (MSI) proposed by Oliver Fiehn *et al*.^61^.

For GC-MS, an in-house program named ‘Feature Fusion’ developed especially for GC-MS data refinement was firstly carried out to discriminate ions derived from the same metabolite. Identification of the metabolite was done by comparison of mass spectra with those available in National Institute of Standards and Technology (NIST) library using a similarity index as a percentage for discrimination from nearest neighbors^62^. Only those peaks with similarity more than 80% were assigned for compound names. Finally, they were further confirmed by comparing the retention time and mass spectra with standard compounds available in our lab.

### Sensitivity analysis

As showed in Figure S5, after differential metabolites screening, sensitivity of cortex and medulla to cisplatin administration was analyzed and validated using RF, heat-map, OPLS-DA score plot, parameter Q^2^, and MCFC methods.

First, to get a general view of the sensitivity difference between cortex and medulla, RF models (R package randomForest) were constructed based on all extracted features from GC-MS and LC-MS. It was used to evaluate the similarity between group C and cisplatin-treated group (i.e. C vs L, C vs M and C vs H) in cortex and medulla^63^,^64^. Based on random stratified sampling, all samples in two groups were divided into training set (two-third) and test set (one-third) randomly. The error rate of test set (ERT) was recorded to evaluate the similarity between the two groups (*e.g.* higher ERT indicates less distinction). In some cases, the high ERT suggested the model itself was not stable enough for further application. But this, in turn, indicated the similarity between groups. For each pair of groups, RF analysis was repeated 1000 times with two levels of argument ‘ntree’ (500 or 1000) and four levels of another primary argument ‘mtry’ (one-fifth, one-fourth, one-third or two-fifth of total number of features). Since ERT was sensitive to sampling and the two primary arguments, random stratified sampling process was repeated 125 times for each pair of arguments. This bootstrap analysis strategy was aimed to eliminate the influence of primary parameters and the limitation of each model. Finally, all ERT values were summarized and the difference between cortex and medulla in corresponding groups was compared.

After metabolite identification, heat-map (MeV, Version 4.6.1, http://www.tm4.org/) with hierarchical cluster analysis results on it was used to exhibit the overall variation trend of metabolites among the four groups (i.e. C, L, M, and H) in cortex and medulla. All identified metabolites in cortex or medulla were included.

Furthermore, based on all those identified differential metabolites, inner relation of variable X (differential metabolites) and Y (groups) were discriminated by OPLS-DA (SIMCA-P software). Here, the horizontal axis represented the first predictive component and vertical axis the first grouping information. From the degree of dispersion of the four groups in X and Y directions, sensitivity difference could be discriminated visually.

After that, OPLS-DA models based on identified metabolites between control group and each cisplatin-treated group were constructed. Notably, OPLS-DA here was traditional, which was different with the above. Here, the horizontal axis represented the first predictive component and vertical axis the first orthogonal component. Parameter Q^2^ that can reflect the predictive ability of the model was compared among different OPLS-DA models. To achieve Q^2^ comparability, all the models were assigned with the same number of predictive and orthogonal components. With Q^2^ as an indicator, sensitivity of cortex and medulla to cisplatin could be evaluated quantitatively.

Metabolic cumulative fold change (MCFC) reflects the cumulative fold change of the metabolites was calculated and used as an aggregated response parameter for metabolic fingerprints. The following process was adapted from the calculation method of metabolic effect level index (MELI)^65^. Firstly, fold changes of the common metabolites between cisplatin-treated groups and control group in cortex and medulla were calculated. Then, fold changes lower than 1.0 were transformed into their reciprocals. Finally, fold changes of all common metabolites were added together and named as MCFC. MCFC of group L, M and H in cortex and medulla was compared to evaluate the degree of cumulative metabolic changes in the two parts.

### Contributing metabolite screening

CAST is a cluster analysis method embedded in MeV software (MeV, Version 4.6.1, http://www.tm4.org/). This method could be used for the clustering of metabolites with strong correlation relationship utilizing Person correlation analysis. In the present study, after sensitivity analysis, two-step CAST strategy was conducted to screen metabolites with large contribution to the sensitivity difference between cortex and medulla (Figure S5). For comparing metabolite variations among cisplatin-treated groups, fold changes of metabolites between cisplatin groups and control group were calculated firstly. Then, a union of metabolites with fold change values were imported to MeV software to perform CAST. The first-step CAST was executed with the correlation coefficient of 0.80, and those metabolites with similar change trends were clustered roughly. Furthermore, the second-step CAST was carried out with the correlation coefficient of 0.98 to cluster metabolites more elaborately. After two-step CAST, metabolites would be classified into several clusters that owned different type of change trend. Heat-map was drawn to exhibit the variation trend of metabolites in each cluster visually. In addition, line plot with fold change value as Y-axis and group as X-axis was utilized to express the variation trend of the representative metabolites in each cluster.

### Pathway analysis

Open database sources, including the KEGG (http://www.genome.jp/kegg/), MetaboAnalyst (www.metaboanalyst.ca), HMDB, and METLIN, were used to identify metabolic pathways related to cisplatin nephrotoxicity.

## Additional Information

**How to cite this article:** Zhang, P. *et al*. Renal Medulla is More Sensitive to Cisplatin than Cortex Revealed by Untargeted Mass Spectrometry-Based Metabolomics in Rats. *Sci. Rep.* **7**, 44804; doi: 10.1038/srep44804 (2017).

**Publisher's note:** Springer Nature remains neutral with regard to jurisdictional claims in published maps and institutional affiliations.

## Supplementary Material

Supplementary Information

Supplementary dataset

## References

[b1] Smith I. & Talbot D. Cisplatin and its analogues in the treatment of advanced breast cancer: a review. Br. J. Cancer 65, 787 (1992). 1616849 10.1038/bjc.1992.169 PMC1977765

[b2] Muggia F. Platinum compounds 30 years after the introduction of cisplatin: implications for the treatment of ovarian cancer. Gynecol. Oncol. 112, 275–281 (2009). 18977023 10.1016/j.ygyno.2008.09.034

[b3] Boulikas T. & Vougiouka M. Recent clinical trials using cisplatin, carboplatin and their combination chemotherapy drugs (review). Oncol. Rep. 11, 559–595 (2004). 14767508

[b4] Hartmann J. T. & Lipp H.-P. Toxicity of platinum compounds. Expert Opin. Pharmacother. 4, 889–901 (2003). 12783586 10.1517/14656566.4.6.889

[b5] Icli F. . Severe vascular toxicity associated with cisplatin-based chemotherapy. Cancer 72, 587–593 (1993). 8319192 10.1002/1097-0142(19930715)72:2<587::aid-cncr2820720242>3.0.co;2-v

[b6] Kounis N. G., Cervellin G. & Lippi G. Cisplatin-induced bradycardia: Cardiac toxicity or cardiac hypersensitivity and Kounis syndrome? Int. J. Cardiol. 202, 817–818 (2016). 26476038 10.1016/j.ijcard.2015.10.027

[b7] Pollera C. F., Ameglio F., Nardi M., Vitelli G. & Marolla P. Cisplatin-induced hepatic toxicity. J. Clin. Oncol. 5, 318–319 (1987). 10.1200/JCO.1987.5.2.318 3806175

[b8] Rabinowits M., Souhami L., Gil R. A., Andrade C. A. & Paiva H. C. Increased pulmonary toxicity with bleomycin and cisplatin chemotherapy combinations. Am. J. Clin. Oncol. 13, 132–138 (1990). 1690503 10.1097/00000421-199004000-00009

[b9] Verschraegen C., Conrad C. A. & Hong W. K. Subacute encephalopathic toxicity of cisplatin. Lung cancer 13, 305–309 (1995). 8719070 10.1016/0169-5002(95)00503-x

[b10] Waissbluth S. & Daniel S. J. Cisplatin-induced ototoxicity: transporters playing a role in cisplatin toxicity. Hear. Res. 299, 37–45 (2013). 23467171 10.1016/j.heares.2013.02.002

[b11] Litterst C. Plasma levels and organ distribution of platinum in the rat, dog, and dog-fish shark following single intravenous administration of cis-dichlorodiammineplatinum (II). J. Clin. Hematol. Oncol. 7, 169–179 (1976).

[b12] Schaeppi U. . cis-Dichlorodiammineplatinum (II)(NSC-119 875): preclinical toxicologic evaluation of intravenous injection in dogs, monkeys and mice. Toxicol. Appl. Pharmacol. 25, 230–241 (1973). 4197634 10.1016/s0041-008x(73)80009-2

[b13] Arany I. & Safirstein R. L. Semin. Nephrol. 23, 460–464 (2003). 13680535 10.1016/s0270-9295(03)00089-5

[b14] Stewart J. D. & Bolt H. M. Cisplatin-induced nephrotoxicity. Arch. Toxicol. 86, 1155–1156 (2012). 22696163 10.1007/s00204-012-0887-2

[b15] Ries F. & Klastersky J. Nephrotoxicity induced by cancer chemotherapy with special emphasis on cisplatin toxicity. Am. J. Kidney Dis. 8, 368–379 (1986). 3538860 10.1016/s0272-6386(86)80112-3

[b16] Gonzalez‐Vitale J. C., Hayes D. M., Cvitkovic E. & Sternberg S. S. The renal pathology in clinical trials of Cis‐platinum (II) diamminedichloride. Cancer 39, 1362–1371 (1977). 851939 10.1002/1097-0142(197704)39:4<1362::aid-cncr2820390403>3.0.co;2-n

[b17] Brady H. R. . Mitochondrial injury: an early event in cisplatin toxicity to renal proximal tubules. Am. J. Physiol. 258, F1181–1187 (1990). 2159714 10.1152/ajprenal.1990.258.5.F1181

[b18] Yonezawa A. . Association between tubular toxicity of cisplatin and expression of organic cation transporter rOCT2 (Slc22a2) in the rat. Biochem. Pharmacol. 70, 1823–1831 (2005). 16242669 10.1016/j.bcp.2005.09.020

[b19] Eljack N. D. . Mechanisms of cell uptake and toxicity of the anticancer drug cisplatin. Metallomics 6, 2126–2133 (2014). 25306996 10.1039/c4mt00238e

[b20] Jeong J. J. . Role of annexin A5 in cisplatin-induced toxicity in renal cells: molecular mechanism of apoptosis. J. Biol. Chem. 289, 2469–2481 (2014). 24318879 10.1074/jbc.M113.450163 PMC3900989

[b21] Wilmes A. . Mechanism of cisplatin proximal tubule toxicity revealed by integrating transcriptomics, proteomics, metabolomics and biokinetics. Toxicol. in Vitro 30, 117–127 (2015). 25450742 10.1016/j.tiv.2014.10.006

[b22] Yousef M. I. & Hussien H. M. Cisplatin-induced renal toxicity via tumor necrosis factor-alpha, interleukin 6, tumor suppressor P53, DNA damage, xanthine oxidase, histological changes, oxidative stress and nitric oxide in rats: protective effect of ginseng. Food Chem. Toxicol. 78, 17–25 (2015). 25640527 10.1016/j.fct.2015.01.014

[b23] Nicholson J., Lindon J. & Holmes E. ‘Metabonomics’: understanding the metabolic responses of living systems to pathophysiological stimuli via multivariate statistical analysis of biological NMR spectroscopic data. Xenobiotica 29, 1181–1189 (1999). 10598751 10.1080/004982599238047

[b24] Nicholson J. K., Connelly J., Lindon J. C. & Holmes E. Metabonomics: a platform for studying drug toxicity and gene function. Nat. Rev. Drug Discovery 1, 153–161 (2002). 12120097 10.1038/nrd728

[b25] Fernández-Peralbo M. A. & Luque de Castro M. D. Preparation of urine samples prior to targeted or untargeted metabolomics mass-spectrometry analysis. Trends Anal. Chem. 41, 75–85 (2012).

[b26] Roberts L. D., Souza A. L., Gerszten R. E. & Clish C. B. Targeted metabolomics. Current protocols in molecular biology Chapter 30 (2012). 10.1002/0471142727.mb3002s98 PMC3334318 22470063

[b27] Lu W., Bennett B. D. & Rabinowitz J. D. Analytical strategies for LC-MS-based targeted metabolomics. J. Chromatogr. B: Anal. Technol. Biomed. Life Sci. 871, 236–242 (2008). 10.1016/j.jchromb.2008.04.031 PMC2607197 18502704

[b28] Lindon J. C., Holmes E. & Nicholson J. K. Metabonomics in pharmaceutical R & D. FEBS J. 274, 1140–1151 (2007). 17298438 10.1111/j.1742-4658.2007.05673.x

[b29] Holmes E., Wilson I. D. & Nicholson J. K. Metabolic phenotyping in health and disease. Cell 134, 714–717 (2008). 18775301 10.1016/j.cell.2008.08.026

[b30] Aldridge B. B. & Rhee K. Y. Microbial metabolomics: innovation, application, insight. Curr. Opin. Microbiol. 19, 90–96 (2014). 25016173 10.1016/j.mib.2014.06.009

[b31] Astarita G. & Langridge J. An Emerging Role for Metabolomics in Nutrition Science. J. Nutrigenet. Nutrigenomics 6, 181–200 (2013). 24009004 10.1159/000354403

[b32] Boudonck K. J. . Discovery of metabolomics biomarkers for early detection of nephrotoxicity. Toxicol. Pathol. 37, 280–292 (2009). 19380839 10.1177/0192623309332992

[b33] Kim M. A., Park S. H., Yang H. J. & Kwon H. N. Identification of urinary biomarkers related to cisplatin-induced acute renal toxicity using NMR-based metabolomics. Biomol. Ther. 19, 38–44 (2011).

[b34] Kwon H. N. . Predicting idiopathic toxicity of cisplatin by a pharmacometabonomic approach. Kidney Int. 79, 529–537 (2011). 20980974 10.1038/ki.2010.440

[b35] Portilla D. . Metabolomic study of cisplatin-induced nephrotoxicity. Kidney Int. 69, 2194–2204 (2006). 16672910 10.1038/sj.ki.5000433

[b36] Portilla D., Schnackenberg L. & Beger R. D. Metabolomics as an extension of proteomic analysis: study of acute kidney injury. Semin. Nephrol. 27, 609–620 (2007). 18061843 10.1016/j.semnephrol.2007.09.006 PMC2684501

[b37] Won A. J. . Discovery of urinary metabolomic biomarkers for early detection of acute kidney injury. Mol. BioSyst. 12, 133–144 (2016). 26566257 10.1039/c5mb00492f

[b38] Zhang P. . Discovery of potential biomarkers with dose and time dependence in cisplatin-induced nephrotoxicity using metabolomics integrated with principal component-based area calculation (PCAC) strategy. Chem. Res. Toxicol. (2016). 10.1021/acs.chemrestox.5b00519 27030963

[b39] Hall J. E. & Guyton A. C. Textbook of medical physiology. 309–310 (Saunders: London,, 2011).

[b40] Dobyan D. C., Levi J., Jacobs C., Kosek J. & Weiner M. W. Mechanism of cis-platinum nephrotoxicity: II. Morphologic observations. J. Pharmacol. Exp. Ther. 213, 551–556 (1980). 7193726

[b41] Townsend D. M. & Hanigan M. H. Inhibition of gamma-glutamyl transpeptidase or cysteine S-conjugate beta-lyase activity blocks the nephrotoxicity of cisplatin in mice. J. Pharmacol. Exp. Ther. 300, 142–148 (2002). 11752109 10.1124/jpet.300.1.142 PMC6522257

[b42] Townsend D. M., Deng M., Zhang L., Lapus M. G. & Hanigan M. H. Metabolism of cisplatin to a nephrotoxin in proximal tubule cells. J. Am. Soc. Nephrol. 14, 1–10 (2003). 12506132 10.1097/01.asn.0000042803.28024.92 PMC6361148

[b43] Pabla N. & Dong Z. Cisplatin nephrotoxicity: mechanisms and renoprotective strategies. Kidney Int. 73, 994–1007 (2008). 18272962 10.1038/sj.ki.5002786

[b44] Karbach U. . Localization of organic cation transporters OCT1 and OCT2 in rat kidney. Am. J. Physiol.-Renal 279, F679–F687 (2000). 10.1152/ajprenal.2000.279.4.F679 10997918

[b45] Zhang J., Zhong L., Zhang M. & Xia Y. Protection effects of procaine on oxidative stress and toxicities of renal cortical slices from rats caused by cisplatin *in vitro*. Arch. Toxicol. 66, 354–358 (1992). 1610298 10.1007/BF01973631

[b46] Tsuruya K. . Direct involvement of the receptor-mediated apoptotic pathways in cisplatin-induced renal tubular cell death. Kidney Int. 63, 72–82 (2003). 12472770 10.1046/j.1523-1755.2003.00709.x

[b47] Cummings B. S. & Schnellmann R. G. Cisplatin-induced renal cell apoptosis: caspase 3-dependent and-independent pathways. J. Pharmacol. Exp. Ther. 302, 8–17 (2002). 12065694 10.1124/jpet.302.1.8

[b48] Zhou M. . Progranulin protects against renal ischemia/reperfusion injury in mice. Kidney Int (2015). 10.1038/ki.2014.403 25607110

[b49] Park J. Y. . Protective Effects of Processed Ginseng and Its Active Ginsenosides on Cisplatin-Induced Nephrotoxicity: *In Vitro* and *in Vivo* Studies. J. Agric. Food Chem. 63, 5964–5969 (2015). 26050847 10.1021/acs.jafc.5b00782

[b50] Pettus B. J., Chalfant C. E. & Hannun Y. A. Ceramide in apoptosis: an overview and current perspectives. Biochim. Biophys. Acta 1585, 114–125 (2002). 12531544 10.1016/s1388-1981(02)00331-1

[b51] Bartke N. & Hannun Y. A. Bioactive sphingolipids: metabolism and function. J. Lipid Res. 50 Suppl, S91–96 (2009). 19017611 10.1194/jlr.R800080-JLR200 PMC2674734

[b52] Abel R. M. . Improved survival from acute renal failure after treatment with intravenous essential L-amino acids and glucose: Results of a prospective, double-blind study. N. Engl. J. Med. 288, 695–699 (1973). 4631743 10.1056/NEJM197304052881401

[b53] Btaiche I. F., Mohammad R. A., Alaniz C. & Mueller B. A. Amino acid requirements in critically ill patients with acute kidney injury treated with continuous renal replacement therapy. Pharmacotherapy 28, 600–613 (2008). 18447659 10.1592/phco.28.5.600

[b54] Wen H. . Identification of Urinary Biomarkers Related to Cisplatin-Induced Acute Renal Toxicity Using NMR-Based Metabolomics. Biomol. Ther. 19, 38–44 (2011).

[b55] Kim H. J. . Glutamine protects against cisplatin-induced nephrotoxicity by decreasing cisplatin accumulation. J. Pharmacol. Sci. 127, 117–126 (2015). 25704027 10.1016/j.jphs.2014.11.009

[b56] Mahran Y. F., Khalifa A. E. & El-Demerdash E. A comparative study of protective mechanisms of glycine and L-arginine against cisplatin-induced nephrotoxicity in rat renal cortical slices. Drug Discoveries Ther. 5, 32–40 (2011). 10.5582/ddt.v5.1.32 22466094

[b57] De Livera A. M. . Normalizing and integrating metabolomics data. Anal. Chem. 84 (2012). 10.1021/ac302748b 23150939

[b58] De Livera A. M. . Statistical methods for handling unwanted variation in metabolomics data. Anal. Chem. 87, 3606–3615 (2015). 25692814 10.1021/ac502439y PMC4544854

[b59] Huang Y. . Discovery of safety biomarkers for realgar in rat urine using UFLC-IT-TOF/MS and 1H NMR based metabolomics. Anal. Bioanal. Chem. 405, 4811–4822 (2013). 23479124 10.1007/s00216-013-6842-0

[b60] Wang J. . Metabolomic study of Chinese medicine Huang Qin decoction as an effective treatment for irinotecan-induced gastrointestinal toxicity. RSC Adv. 5, 26420–26429 (2015).

[b61] Members M. S. I. B. . The metabolomics standards initiative. Nat. Biotechnol. 25, 846–848 (2007). 17687353 10.1038/nbt0807-846b

[b62] Schauer N. . GC-MS libraries for the rapid identification of metabolites in complex biological samples. FEBS Lett. 579, 1332–1337 (2005). 15733837 10.1016/j.febslet.2005.01.029

[b63] Breiman L. Random forests. Mach. Learn. 45, 5–32 (2001).

[b64] Liaw A. & Wiener M. Classification and Regression by randomForest. R News 2, 18–22 (2002).

[b65] Riedl J. . Metabolic Effect Level Index Links Multivariate Metabolic Fingerprints to Ecotoxicological Effect Assessment. Environ. Sci. Technol. 49, 8096–8104 (2015). 26020363 10.1021/acs.est.5b01386

