# Supplementary material for: Renal Medulla is More Sensitive to Cisplatin than Cortex Revealed by Untargeted Mass Spectrometry-Based Metabolomics in Rats
Source: Sci Rep. 2017 Mar 16;7:44804. doi: 10.1038/srep44804 (PMC5353697; doi:10.1038/srep44804)
Supplement: Supplementary Information [file srep44804-s1.pdf]

Supporting Information

Renal Medulla is More Sensitive to Cisplatin than Cortex Revealed by Untargeted Mass Spectrometry-Based Metabolomics in Rats

Pei Zhang[1,2,3], Jia-Qing Chen[1,2,3], Wan-Qiu Huang[1,2,3], Wei Li[1,2,3], Yin Huang[1,2,3], Zun-Jian Zhang[1,2,3*] and Feng-Guo Xu[1,2,3*]

[1] Key Laboratory of Drug Quality Control and Pharmacovigilance (Ministry of Education), China Pharmaceutical University, Nanjing 210009, P. R. China.

[2] Jiangsu Key Laboratory of Drug Screening, China Pharmaceutical University, Nanjing 210009, P. R. China.

[3] State Key Laboratory of Natural Medicine, China Pharmaceutical University, Nanjing 210009, P. R. China.

*** Corresponding author:**

Feng-Guo Xu,

Key Laboratory of Drug Quality Control and Pharmacovigilance (China Pharmaceutical University), Ministry of Education, Tongjiaxiang No. 24, Nanjing 210009, China;

Tel/Fax: +86 025 83271021; E-mail: fengguoxu@gmail.com

Zun-Jian Zhang,

Key Laboratory of Drug Quality Control and Pharmacovigilance (China Pharmaceutical University), Ministry of Education, Tongjiaxiang No. 24, Nanjing 210009, China;

Tel/Fax: +86 025 83271454; E-mail: zunjianzhangcpu@hotmail.com

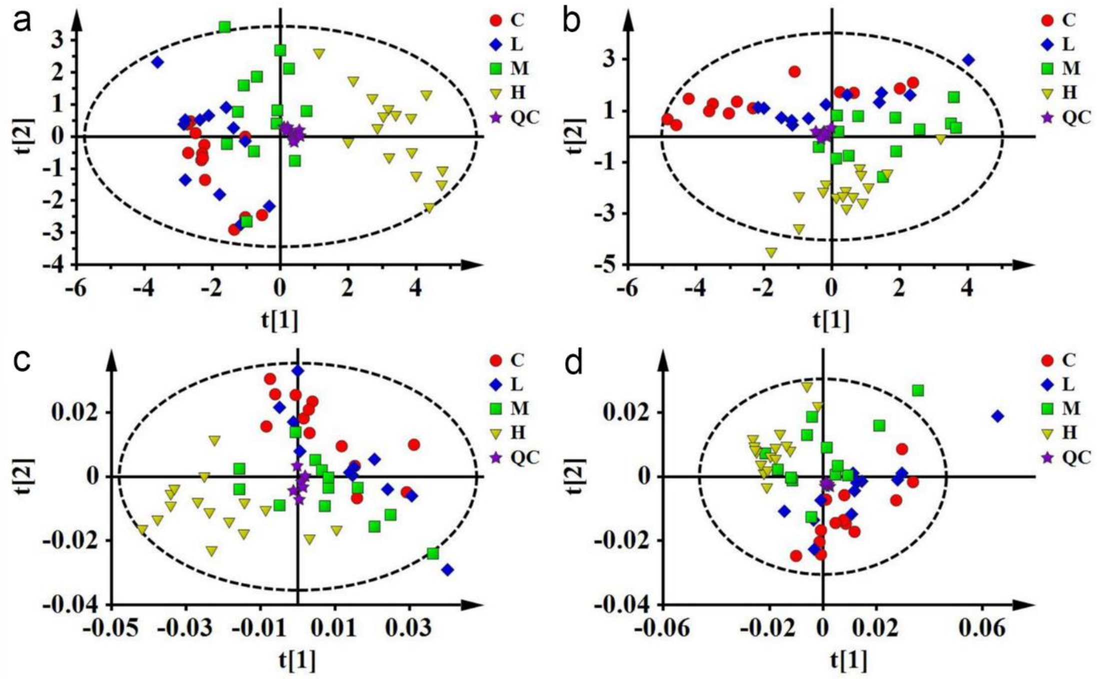

Figure S1. Data quality evaluation for untargeted metabolomics using PCA. PCA score plot of (a) QCs and cortex samples based on GC-MS data, model parameter: R^2X=0.48, Q^2=0.366; (b) QCs and medulla samples based on GC-MS data, model parameter: R^2X=0.671, Q^2=0.568; (c) QCs and cortex samples based on LC-MS data, model parameter: R^2X=0.593, Q^2=0.307, and (d) QCs and medulla samples based on LC-MS data, model parameter: R^2X=0.629, Q^2=0.297.

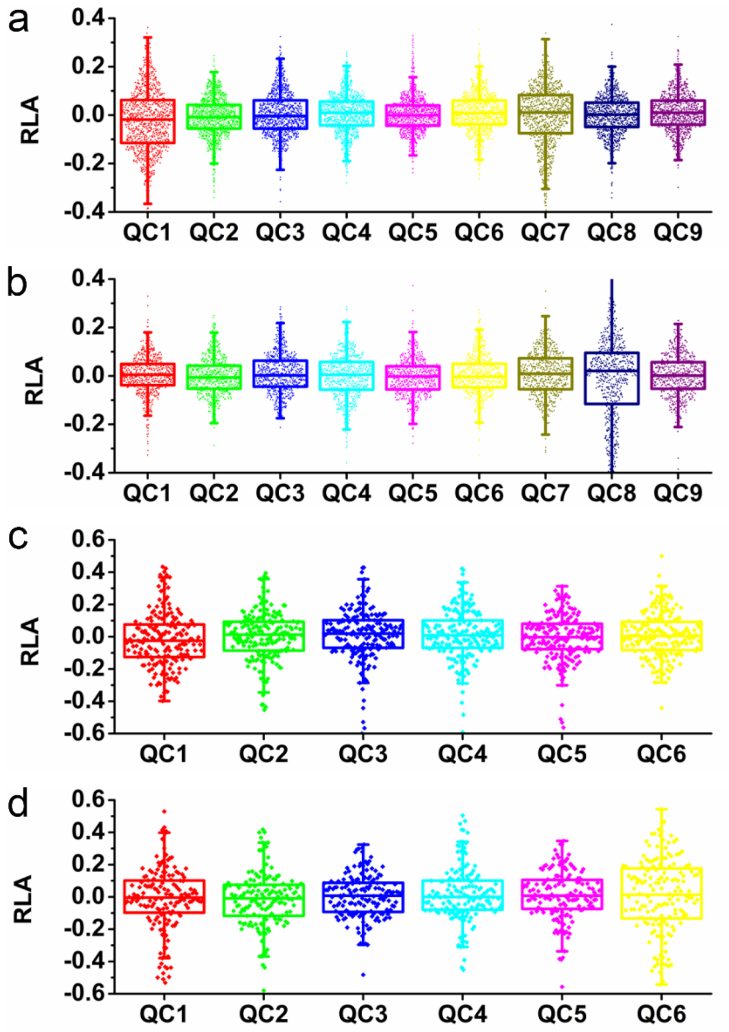

Figure S2. Data quality evaluation for untargeted metabolomics using RLA plot. RLA plot of (a) QCs of cortex in GC-MS analysis; (b) QCs of medulla in GC-MS analysis; (c) QCs of cortex in LC-MS analysis, and (d) QCs of medulla in LC-MS analysis.

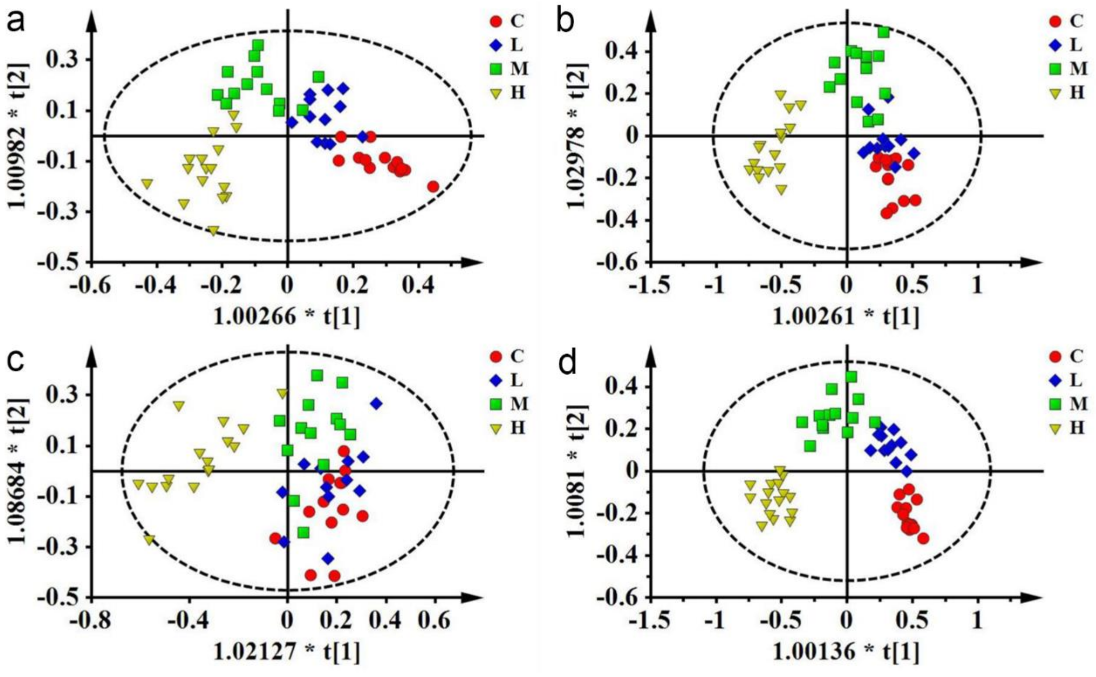

Figure S3. OPLS-DA score plots of (A) cortex samples based on GC-MS data; (B) cortex samples based on LC-MS data; (C) medulla samples based on GC-MS data and (D) medulla samples based on LC-MS data.

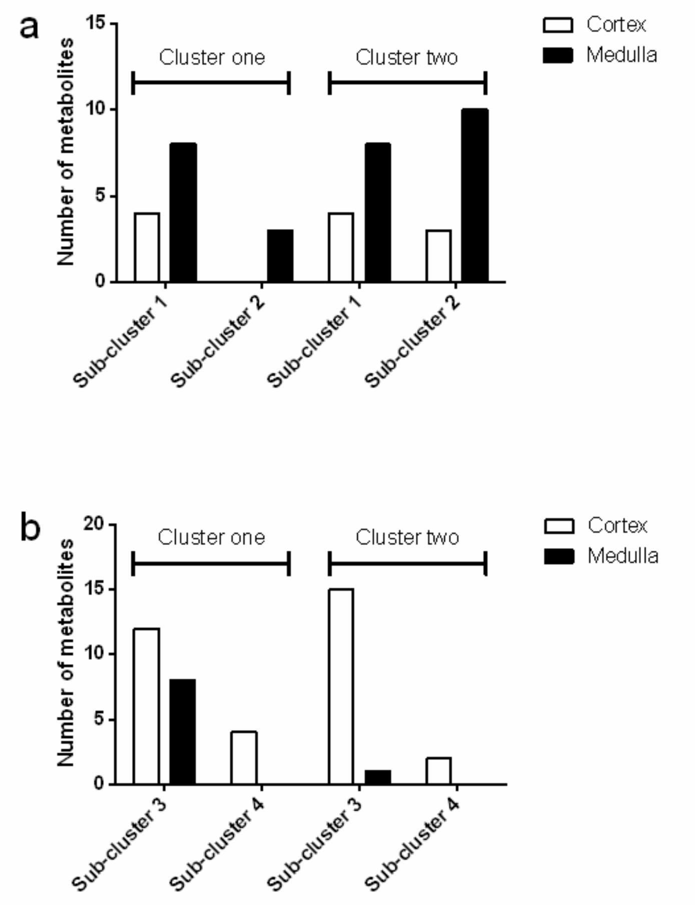

Figure S4 Histogram of the number of metabolites. (a) Metabolites in sub-cluster 1 and 2 withine cluster one and two; (b) Metabolites in sub-cluster 3 and 4 within cluster one and two.

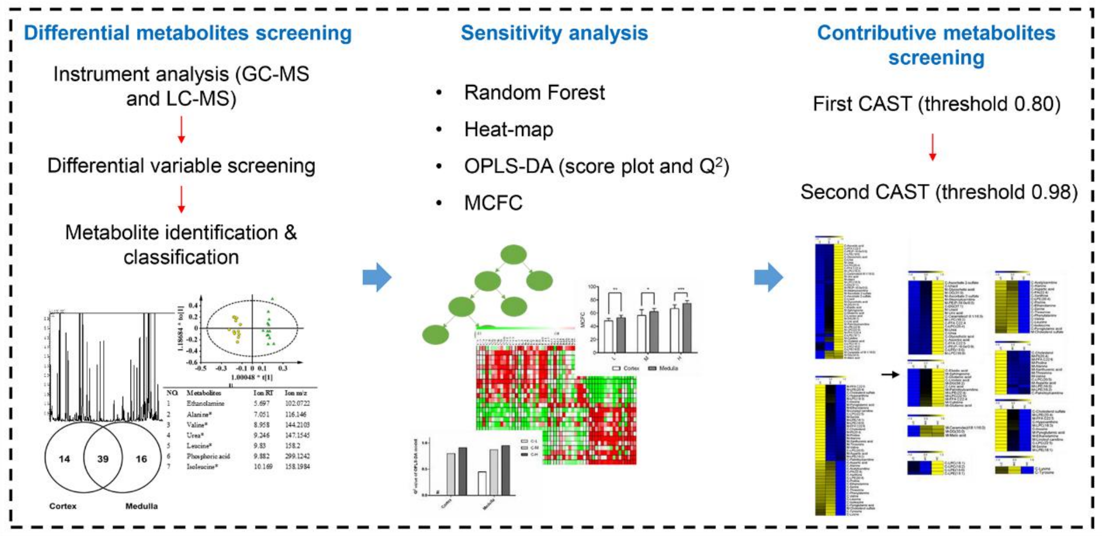

Figure S5. Strategy for differential metabolites screening, sensitivity analysis and contributory metabolites screening.
